# The dynamic system of parental work of care for children with special health care needs: A conceptual model to guide quality improvement efforts

**DOI:** 10.1186/1471-2431-11-95

**Published:** 2011-10-25

**Authors:** Kari R Hexem, Abigail M Bosk, Chris Feudtner

**Affiliations:** 1 PolicyLab and The Department of Medical Ethics, The Children's Hospital of Philadelphia, Philadelphia PA, USA

## Abstract

**Background:**

The work of care for parents of children with complex special health care needs may be increasing, while excessive work demands may erode the quality of care. We sought to summarize knowledge and develop a general conceptual model of the work of care.

**Methods:**

Systematic review of peer-reviewed journal articles that focused on parents of children with special health care needs and addressed factors related to the physical and emotional work of providing care for these children. From the large pool of eligible articles, we selected articles in a randomized sequence, using qualitative techniques to identify the conceptual components of the work of care and their relationship to the family system.

**Results:**

The work of care for a child with special health care needs occurs within a dynamic system that comprises 5 core components: (1) performance of *tasks *such as monitoring symptoms or administering treatments, (2) the occurrence of various *events *and the pursuit of *valued outcomes *regarding the child's physical health, the parent's mental health, or other attributes of the child or family, (3) operating with available *resources *and within certain *constraints *(4) over the passage of *time*, (5) while mentally representing or *depicting *the ever-changing situation and *detecting *possible problems and opportunities. These components interact, some with simple cause-effect relationships and others with more complex interdependencies.

**Conclusions:**

The work of care affecting the health of children with special health care needs and their families can best be understood, studied, and managed as a multilevel complex system.

## Background

Medical care advances have dramatically reduced pediatric morbidity and mortality - from formerly premature infants with severe chronic lung disease requiring supplemental oxygen and assisted ventilation, through to adolescents with treatment-refractory epilepsy or myriad other medical conditions - while creating new challenges for children and families [1,2]. In the United States, approximately 13 to 20% of households have an infant, child, or adolescent with a special health care need (CSHCN) [3]. The mounting dependency on medical technology, reliance on multitudes of medications, and intense use of medical services place increased demands on parents. At the same time, parents must also navigate the complicated systems of health insurance and childhood education, while attending to other responsibilities including maintaining their own mental and physical health and that of their families, and wrestling with the larger existential questions posed by their child's illness.

While these dimensions of the experience of illness or disability are often referred to as "caregiving" or the "burden of care", the phrase "work of care" (WOC) specifies the physical and mental efforts of specific tasks in which parents engage, while avoiding the conflation in the term "caregiving" of both a person who is a caregiver and a set of actions that constitute caregiving, and the negative and potentially biased emotive connotations of "burden". While physicians, nurses, and the broad health care system are certainly important to the health and wellbeing of CSHCN, and at various times during a child's life (such as during a hospitalization) may share in performing the tasks involved in caring for the child, the WOC is chiefly performed by the patient and family. Parental WOC is too often an overlooked component when assessing the quality of care structure, processes, and outcomes for CSHCN [4]. In 2003, an Institute of Medicine Report identified 7 key processes of care for CSHCN: care planning, use of preventive services, access to specialists, ancillary services, mental health and dental services, and care coordination [5]. The report failed to address, however, the cumulative and interrelated effects of these processes - which is to say, how the WOC operates within a multi-component dynamic system.

To synthesize and extend our current understanding of the parental WOC for CSHCN, we created a conceptual model via a systematic review of the published literature, aiming to provide a representative synopsis of both empirical findings and perspectives, which can then be used to "clarify, describe, and organize ideas" about how to improve the quality of care for CSHCN [6]. As an initial point of entry into the topic, we located the WOC concept at the intersection of the theoretical frameworks of the sociology of work[7], the psychology of coping [8], and the emerging field of complex systems [9,10]. Taken separately, these frameworks pose interesting and important questions, such as: Why are certain tasks identified as valuable and others are not, and why are specific tasks assigned to specific persons? How do people cope with stressful life events, and how do they use resources in other areas of their lives to do this? How do people's responses to events shape, in ways both predicable and unpredictable, future events? When combined, a conceptual model synthesizing these three frameworks both specifies the tasks inherent to the WOC while sketching an integrated model of how the dynamic WOC system operates as parents attempt to mount an adaptive response to the challenging circumstances of parenting a child with special health care needs.

## Methods

**Figure 1:**
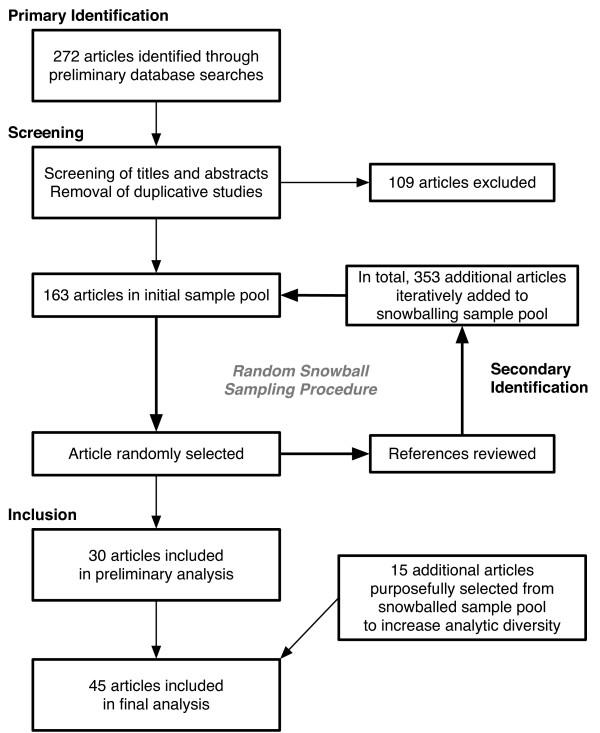
Procedure for systematic identification and selection of included articles.

We performed a systematic literature review in multiple databases, and also reviewed the reference sections of articles randomly selected for analysis (Table 1 provides additional information regarding the search [11]). Inclusion criteria were purposefully broad, aiming to capture the range of research questions and methods. We excluded articles focused on non-parental caregivers (such as nurses or home health aids, but including other parental adults such as foster parents or extended family members), caregivers of aging parents, or articles on bereaved parents whose children had previously died. The following databases were reviewed: PubMed, MEDLINE, PsycINFO (Psychological Abstracts), and CINAHL (Cumulative Index to Nursing and Allied Health Literature). Combining randomization with snowball sampling approaches, our strategy, conducted by 2 independent reviewers, proceeded as follows (Figure 1): Using the specified search terms and eligibility criteria, 272 articles were initially retrieved from the databases. We reviewed the titles and abstracts, culling the initial set down to 163 articles. The titles of these pertinent articles were listed in alphabetical order and enumerated from first to last (N_1_). Using a random number generator (available at http://www.random.org), a number was selected between 1 and N_1_, corresponding to a unique article. The corresponding article was read, relevant data or concepts were abstracted, and the article's references were reviewed (by reading titles and abstracts), with all newly identified relevant articles added to the list of pertinent articles. The new augmented list was re-enumerated from 1 to N_2_; a second number was randomly selected between 1 and N_2_; and the second article was identified, the results analyzed and abstracted, and the references reviewed. This procedure was repeated until the review process had reached a point of thematic saturation, where additional successive articles were no longer adding new information or concepts [12].

**Table 1:** Systematic review methodology

Methods topics	Description	Rationale
Eligibility Criteria		
Years	All	To assess change in patterns of reporting over time.
Language	English only	Study authors were only fluent in English.
Publication status	Peer reviewed journals	Peer review set minimum criteria for quality, journals used as primary medium for communication of information.
Information sources		
Databases	PUBMED, PSYCINFO, CINAHL, and SOCIOLOGICAL ABSRACTS	Multiple databases were selected to provide access to a breadth of journals.
References	Review of reference sections	Provided additional articles not found in database searches.
Search	PUBMED example 1: "work+care+burden+pediatric" 2: "caregiving+coping+child+chronic" 3: "caring+children+chronic+disease+parents" 4: "caregivers+role+strain+child"	Multiple searches using different terms revealed different journal articles to review.
Study selection	See figure 1	
Data collection process	Data extracted from 30 journal articles chosen in random order, and 15 additional articles purposefully selected to increase diversity of sample	Used to prevent bias while at the same time sampling from entire population of articles.
Data items	See Tables 3 and 4	Code list generated from qualitative methods based on Grounded Theory (Strauss & Corbin 1987).
Summary measures	See Tables 3 and 4	Categories based on code list.
Synthesis of results	Theoretical model used to organize codes/categories	Theoretical model based on data as well as theories of work, coping, and complex systems.
Risk of bias across studies	"Medicalization" of work of care in peer review journals, exclusion of lay literature	Journal audience is comprised of medical and research personnel.

With the above procedure, we ultimately identified 516 published articles in English, which were analyzed in random order until thematic saturation was achieved (n = 30). Fifteen additional articles were purposefully sampled from the titles of the remaining 486 references to increase the representation of longitudinal observational studies (n = 5), intervention studies (n = 5), and studies focused on minority populations (n = 5), bringing the total number included in this review to 45 papers.

Using an iterative process, a preliminary list of WOC codes was created and revised following the guidelines of grounded theory [13]. We used this coding strategy to systematically identify and organize the components of the work of care into a conceptual model, based on numerous conversations among the authors, feedback from colleagues who work extensively with CSHCN, and concepts from the frameworks of the sociologies of work, coping, and complex systems.

## Results

Articles addressing parental WOC associated with CSCHN were published in 199 different journals devoted to pediatrics and allied disciplines, with 123 journals publishing one article only and 9 journals publishing more than 10 articles (Table 2). Most studies were qualitative research studies (70%) or literature reviews (26%). The majority of research studies were cross-sectional and observational (70%).

**Table 2:** Study information for articles retrieved using databases, iterative review of each paper's references, and purposefully

	N
**Total sample pool of 516 articles from 199 journals**	
Mean articles per journal	2.6
Number of journals with one article only	123
Number of journals with 10 or more articles	9
**Randomly selected 30 articles from 24 journals**	**N (%)**
Retrieved from	
PUBMED	14 (47%)
Reference sections of articles	9 (30%)
Other databases	7 (23%)
Study type	
Qualitative (Interviews, Focus Groups, Ethnography)	13 (43%)
Literature reviews^+^	8 (26%)
Quantitative (Survey, Time diaries)	7 (20%)
Mixed methods	2 (7%)
Randomized Intervention Studies	0
Study period	
Cross-sectional or one visit	21 (70%)
Longitudinal or multiple visits	1 (3%)
N/a (literature review)	8 (27)
Contained visual model	
Yes	5 (17%)
No	25 (83%)
Disease type	
Unspecified (CSCHN, Chronic Illness, Disability)	16 (53%)
Asthma	3 (10%)
Epilepsy	2 (7%)
Assisted by medical technology	2 (7%)
Type 1 Diabetes	2 (7%)
Other*	5 (17%)
Country of study site	
United States	14 (47%)
Australia, Canada, U.K.	11 (37%)
Other**	5 (17%)
Gender of parent-participants	
Mothers only	5 (17%)
Mothers and fathers eligible, greater than 55% mothers	7 (23%)
Mothers and fathers eligible, less than or equal to 55% mothers	3 (10%)
Fathers only	2 (7%)
Unspecified	5 (17%)
N/a (literature review)	8 (27%)
Addressed racial or ethnic minorities	
Yes	1 (3%)
No	29 (97%)
**Purposefully selected 15 additional articles from 10 journals**	
Longitudinal observational studies	5 (33%)
Intervention studies	5 (33%)
Addressed racial, ethnic, or very poor minorities	5 (33%)

^+^Included only 1 Literature Review with explicit search criteria. * Other disease includes 1 of each of the following: Anorexia, Cancer, Juvenile Rheumatoid Arthritis, Schizophrenia, and Infants At-Risk of SIDS.

** Other country includes 1 of each of the following: Germany, New Zealand, Sweden, Switzerland, and 1 study comparing U.S. and Icelandic populations

### Work of care components

**Figure 2:**
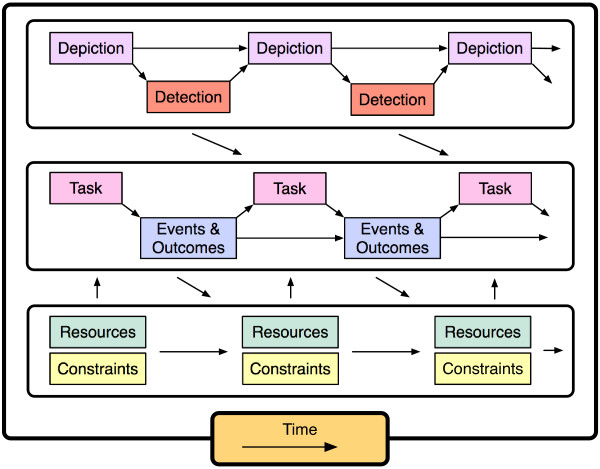
Core components of the dynamic system of parental work of care.

We organized our findings within the framework of a conceptual model developed over the course of performing this review. Figure 2 illustrates the proposed relationships among the following components: (1) performance of tasks, (2) occurrence of events and pursuit of valued outcomes, (3), use of resources and limits of constraints, (4) passage of time, and (5) mentally depicting the situation and detecting problems and opportunities.

#### (1) Performance of Tasks

The work of care, on the surface, is all about tasks: the things parents *do *to achieve the aims of caring for their child, their family, and themselves. These tasks vary greatly, from medication management for a child with asthma [14-16] to completing the extensive paperwork required to receive insurance coverage for a child on a ventilator receiving care at home [17]. Tasks pertaining to the care of the child also include communicating and collaborating with medical professionals [18-20], crisis care, intensive care and emergency room visits [19-23], decision-making about which tasks to take on and who should perform them [15,17,19,24], seeking medical information, and learning new skills [17-20,22,24]. Parents must also continue to perform the regular tasks of parenting, such as providing emotional support to the child and helping him or her with normal developmental needs [19,24-28].

Additionally, parents engage in a variety of WOC tasks pertaining to the family and caregiver including creating daily routines and maintaining a sense of "normal" life [17,21,29,30], caring for siblings [17,29,31-35], managing paid employment [31-34,36] and household chores [17,22,32,33,37], helping and supporting each other [15,19,22,23,29], and communicating skillfully with other family members about the situation [19,38].

The parent must also perform WOC tasks for her- or himself that include both self-care and finding support. Self-care, which in the literature is focused predominantly on emotional management [19,22,23,25,26,30,37-39], should also include attention to diet, exercise and relaxation [22,32,33,40], and may include personal spiritual or religious practices such as prayer or meditation [19,38,40,41]. Finding support includes both maintaining communication with friends and family [19,26,32,33,42,43], and seeking the support of other families in similar situations through formal support groups as well as internet-based groups [17-19,26,33,38,44]. The complete list of WOC tasks is presented in Table 3.

**Table 3:** Work of Care Tasks

Category	Codes	Papers
**CHILD WITH SPECIAL HEALTH CARE NEEDS (CSHCN) CARE WORK**		
Medical tasks	Medications and care of technical devices	[15,18,21,24,25,30,37,52,55,56]
Management	Overall management	[15,18,19,22,23,26,38,39,56]
	Collaboration with medical professionals	[18-20]
	Bureaucracy/paperwork	[17,19,22,50]
	Crisis care and emergency room visits	[19,21-23,57]
Monitoring	Constant awareness of child's health status	[15,18,22,25,38,42,52,58]
Decision Making	Deciding which tasks to take on, and which roles.	[15,17,19,24]
Education	Seeking medical information/skills training	[17-20,22,24,46]
Parenting tasks	Emotional support and developmental needs	[19,20,24-28]
**FAMILY CARE WORK**		
Day-to-day	Daily routines and "normal" life, time management	[17-19,21-23,26,29,30,32,33,46,58]
Division of labor	Care of siblings	[17,22,23,29,31,32,35,38,52,58]
	Employment	[31-34,36]
	Household chores	[17,22,32,33,37]
	Helping and supporting each other	[15,19,22,23,26,29,30,59]
Communication	Talking with other family members about situation	[19,38,43]
**PARENTAL SELF CARE WORK**		
Self-care	Emotional management	[19,22,23,25,26,30,37-41,43]
	Diet, exercise, and relaxation	[22,32,33,40]
	Personal religious practice (i.e. prayer)	[19,38,40,41]
Finding support	Maintaining communication with friends	[19,26,32,33,42,57]
	Support of other families in similar situations	[17-19,26,33,38,44,46]
**SOCIETAL WORK**		
Advocacy	Educating others	[19,26]

Individual tasks are not performed in a vacuum. A task may be combined synergistically with other tasks, such as when the completion of one task either aids in the performance of another, or reduces the second task's importance. For example, parents taught the task of administering a rectal medication for seizures in their children with epilepsy had fewer ER visits than parents who were not taught the procedure [21]. Since ER visits may also be considered a task, the introduction of the former task had the ability to diminish the latter. Tasks, however, may also combine antagonistically when different tasks require large amounts of time and energy. For example, parents often have difficulty simultaneously caring for their child and maintaining paid employment [36].

#### (2) Occurrence of Events and Pursuit of Outcomes

Tasks do not affect other tasks directly. Rather, the performance of each task is followed by events or results in an outcome that then changes the situation, creating a feedback loop that changes or creates subsequent tasks. Events may be expected, such as the progression of issues associated with type 1 diabetes over time [30], or unexpected, such as managing acute emergencies during working hours [36] or being laid off from work, and may or may not be causally related to preceding task performance, and the occurrence of events may be difficult to predict in the immediate future.

In most instances, the primary motivational structure for the WOC is the pursuit of desired outcomes, which may be tied directly to improved health status for the child (such as the pursuit of a lower blood sugar for children with diabetes in order to prevent diabetes-related complications [30,37]), or more generally to improvements in the quality of life for the child [21,31] or parent (which could come about, for example, by a reduction in the stress associated with the WOC [31]), or even to a desire to adhere to medical advice, (for example in the management of adolescent asthma) [15]. Precisely which outcomes are being sought is due in large part to the process of depiction, described below.

#### (3) Influences of Resources/Constraints

Resources are elements that parents draw on in order to function efficiently, and have a Janus-like quality of enabling some actions while also imposing constraints due to the nature or quantity of the resources (Table 4). Resources/constraints can emanate directly from the child, or the parent, or be aspects of the larger situation. Differences in how groups of people respond to similar situations may be attributable to both current and historical resources/constraints. As with tasks, each potential resource/constraint interacts with other resources/constraints.

**Table 4:** Work of Care Resources/Constraints

Category	Codes	Papers
**CHILD**		
Gender	How child's gender affects the situation	[14,17,37]
Disease	Diagnosis and prognosis	[24,32,37,41,45,60]
	Severity, symptoms, and child's quality of life	[14,17,20,31,32,41,45,51,56]
	Episodic quality of illness and uncertainty	[41,42,49,57]
Medical care	Type of technology or equipment	[31,42,45,52,58]
	Frequency of treatments	[32,56]^42^
Age	Newborns, children, and adolescents	[14,15,17,33,45]
	Transitioning to Adult Care	[24,27]
Behavior	Cognitive and emotional function/expression	[14,32,45]
	Functional ability/activity limitations	[14,32,45,61]
Location	Home, hospital, or elsewhere	[14,17,20,25,31,37,47,51,58]
**PARENT**		
Gender roles	How the roles of mothers and fathers differ	[14,30,31,37,41,49,52,56,59,61]
Mental health	Emotions, quality of life, and stress	[14,20,31,32,34,36-38,40,41,43,45,51,52,55,59,61,62]
Personality	Hardiness, self-esteem, and coping style	[56,57,59,61,63]
Physical health	Sleep, immune function	[33,55,58]
Knowledge	Medical and parenting skills and experience	[21,47]
	Education level	[34,39]
Social support	Availability of friends and family	[17,31,32,57-59,61,63]
**FAMILY**		
Family structure	Family cohesion, including marital dynamics	[35,38,56,58,59,63]
	Single parents	[14,31,34]
	Step parents and foster parents	[45,49]
	Siblings	[14,32,35,52,58]
Finances	Employment, income, and expenses	[17,31,32,34,36,37,47,55,56,58,60]
	Insurance and eligibility for services	[17,45,49,51,60]
**SOCIETY**		
Minority status	Race, ethnicity, language, culture, and SES	[15,28,47,50,51]
Geographic locale	Different regions, countries. Immobility.	[45,47,50,56,61]
Attitudes and norms	Disability and disease in childhood	[25,28]
	Parental responsibility and gender norms	[24,25,28,41,47,49]
Health services	Availability of care facilities and providers	[23,24,31,50]
	Awareness of available services	[32,45]
Political system	Government policies and funding	[17,31,34,36,45,47,50,56,58]

To illustrate the category of resources/constraints, consider several examples. First, the age of children alters many of the WOC tasks. Newborn children are small and easier to hold, bathe, and transport, but also present constraints because of uncertain diagnoses, fragility associated with being very young, and the need for medical technologies that are appropriate for children of such a small size [42]. If the child lives until young adulthood, he or she is usually better able to care for himself or herself, both in terms of medication management and emotionally. Another set of problems, however, may emerge, both in terms of increasing behavioral problems [15,27] and limited health services availability [24,45].

Second, across the age spectrum, medical technologies aim to allow families to accomplish the major tasks of improving the child's medical stability and quality of life. From feeding and breathing tubes to wheelchairs, dialysis machines to cardiac monitors, each technology provides specific benefits, and each has its tasks: of complex cleaning and maintenance, and of emotional acceptance [31,46,47]. If the tasks associated with medical technologies are too great, medical technologies may cease to be seen as a valuable resource for families, and instead may become a constraint. In this way, it is possible to see how the tasks associated with certain resources can make those resources less desirable.

Third, both gender and mental health of the parents are important and interrelated resource/constraints. In general, mothers and fathers experience the work of care differently [30,31,37]. Mothers are usually the primary caregivers, and are more likely to feel that, if one parent needs to stay home to care for the sick child, they are responsible. In taking on the primary caregiver role, mothers are less likely to be employed outside the home, and within the home often focus on the tasks central to the child, leaving others to deal with household chores and siblings [31,33,48]. When work of care tasks increase and are more difficult, mothers may become more anxious or depressed [48]. But mothers also report positive mental health states, such as love and appreciation [39]. Fathers' roles and emotional responses may also be gender specific. Fathers report struggling with role strain, in particular the 'provider' role, and role confusion as tasks and responsibilities are divided within the household, and fathers may also experience the same emotional pain and struggles as mothers while receiving less attention and support [41]. Additionally, fathers try to be strong for others, which reduces their support seeking from spouses and others [30]. The presence of stepfathers [49] and foster parents [45], however, warn against overly simple generalizations regarding gender roles and role-related differences.

Finally, minority populations may (emphasizing the caveat that published studies are relatively limited) assign different meanings to their child's illness [28], have a greater acceptance of the labor required for the WOC [50,51], and simultaneously have greater difficulty establishing relationships with health care providers, elements that may be compounded by language barriers[50].

#### (4) The Constraints and Passage of Time

Time acts as a constraint to the number of tasks a family can accomplish, while also serving as the ever-changing backdrop to the WOC, leading to predictable and unpredictable, controllable and uncontrollable, outcomes and events [30,33,52]. Time restricts the availability to pursue other activities, either with the family or alone. In addition, over time, children - including ill children - and families grow and develop. These developmental changes continually alter and complicate the WOC in subtle and overt ways [53].

#### (5) Depicting Situations and Detecting Problems and Opportunities

A parent's interpretation or understanding of a child's medical situation and changes in that situation may or may not be conscious, yet it provides the foundation from which a parent executes particular tasks. Essentially, the meaning that parents give to their child's situation defines the WOC in important ways, and is likely to be related (in critical ways) to their beliefs about the large existential questions their child's illness presents [54]. For example, parents may question and try to understand their situation, and struggle to accept their child's condition [19,22,25,43]. Or parents may question their child's diagnosis and have their own interpretation of the illness's meaning [25,28,38]. Parents may also evaluate their situation on the basis of how 'in control' they feel, and become involved in tasks of care in such a way as to increase their level of control [18,19,41]. Another example of a depiction is when parents stress the positive, and try to stay optimistic about their situation [19,39,41]. The process by which families identify difficulties or problems and how they choose to focus on certain aspects while at the same time ignoring others, depends on the families' overall depiction of the situation.

Framed by their depiction of the situation, families detect problems. Often, detection is accomplished by constant monitoring [18,22,42]. If problems are not detected, care may inadequately meet the needs of the children. For example, in a study of parents of adolescents with asthma, parents perceived their adolescents to be more competent at self-care than the adolescents actually were [15]. How parents depict their situation and detect problems within it will affect the tasks they perform, and may be influenced by resources or lack thereof that aid or hinder these efforts.

### Complex causal relationships and emergent phenomena

While relatively simple cause-effect relationships exist in the work of care - such as if a medicine dosage is either missed or overdosed then an untoward event can occur - the interrelationships among elements in each category with the WOC system can create downward or upward spirals of cascading events. Positive feedback loops, for instance, can potentially produce a sequence whereby the WOC results in a parent curtailing and then quitting work, with consequently less income and greater financial constraints, greater stress and reduced task efficiency, and poorer health status [31]. Similar feedback loops can operate in the other direction, for example in the COPE (Creating Opportunities for Parental Empowerment) intervention, wherein a shift in parental knowledge and skills can lead to stronger beliefs in their ability to manage the situation, and therefore less parental stress and fewer subsequent behavioral issues in the child [20]. Through the interaction of these relationships, the WOC acquires characteristics of a multilevel complex adaptive system, wherein the pursuit of specific health outcomes may or may not be aligned with the system's requirement for overall equilibrium [9,10].

## Discussion

Across several domains in the published literature, multiple studies delineate the various components of the dynamic parental WOC system for CSHCN. We assert that in conceptually uniting this body of research, work is the most useful underlying construct. Work emphasizes not only the *content *of the multiple components of care, but also the *process *of how those core components interact in a dynamic system - a system that affects the health and wellbeing of these children and their families in ways that are critically important, but not yet fully understood. The dynamic WOC system framework established here is intended to sharpen the analytic focus on the complex, systemic interaction of these components of care. Through this framework, we hope to provide a map for more clearly understanding the work of care, and ways in which we can support both families and health care teams as they address the needs of children with special health care needs.

### Strengths and limitations of our review

Our sample was based on a systematic review of the medical, nursing, psychological, and sociological literature on topics related to the WOC for children with special health care needs, and evaluated using a thorough randomization approach. Our analysis was organized according to a conceptual model with well-defined categories and directional arrows identifying plausible causal mechanisms. Our study had the major limitation, however, of focusing only on the professional literature to the exclusion of the numerous parent and lay resources that address WOC issues. We chose this strategy in order to better understand how the healthcare community, in particular, has addressed the parental WOC.

### WOC and improving the quality of care

If the work of care for parents of CSHCN is, as our review and conceptual model suggests, a complex multilevel adaptive system, then efforts to improve the quality of care received by these children, aiming ultimately to improve their outcomes, must appreciate and grapple with several features of such systems. Chief among these are potential non-linear interdependencies among components of the work of care, which caution us that improvements in certain tasks of the work of care may affect the performance of other tasks, for better or for worse, either in a graded manner (more time performing one task results in an equivalent lessening of time performing another task) or at certain "tipping points" (when the performance of a task goes from barely sufficient to morbidly insufficient)[10]. Observational and interventional studies of parental WOC need to consider measurements from this systems perspective, including not only several process measures for different core components (such as presence of a care plan or a physician checklist), but also aspects of parental self-care (mental health screenings, coping mechanisms) and child outcomes (functional status or quality of life in various domains as well as morbidity and mortality). The system (including the patient and family's socioeconomic status and cultural context) also changes and adapts over time, suggesting both a need for longitudinal studies and for studies to consider potential effect mediators and moderators in both study design and sampling strategies.

## Conclusions

The work of care warrants greater attention and investigation. Three topics in particular merit rigorous study. First, we need to improve our epidemiologic and sociological understanding of the WOC system. Is the volume of parental WOC truly increasing for the population of parents of CSHCN? What are the most prevalent or influential tasks, and how do key resources or constraints affect task performance? What are the most informative ways to gather and analyze dynamic WOC system data (such as prospective cohort studies and structural equation or non-parametric dynamic system modeling)? Second, we should examine how increases in the parental WOC load relate to changes in work efficiency, and potentially diminishing returns or errors of commission or omission, and develop methods to feasibly monitor and optimize workload. Third, we need to study the occupational health and safety of parents, and the role and impact of broader societal support (through programs such as family medical leave, health insurance benefits that include nursing and respite, and social security income supplementation). These are some of the tasks we need to perform to create new resources to better manage the work of care and improve the outcomes for children with CSHCN and their families.

## Competing interests

The authors declare that they have no competing interests.

## Authors' contributions

KRH participated in the design of the study, performed the data retrieval, analyzed and interpreted the data, drafted sections of the manuscript, revised the manuscript for key intellectual content, read and approved the final manuscript. AMB participated in the design of the study, performed the data retrieval, analyzed and interpreted the data, revised the manuscript for key intellectual content, read and approved the final manuscript. CF participated in the design of the study, analyzed and interpreted the data, drafted sections of the manuscript, revised the manuscript for key intellectual content, read and approved the final manuscript.

## Pre-publication history

The pre-publication history for this paper can be accessed here:

http://www.biomedcentral.com/1471-2431/11/95/prepub
